# A New Biomarker of Fecal Bacteria for Non-Invasive Diagnosis of Colorectal Cancer

**DOI:** 10.3389/fcimb.2021.744049

**Published:** 2021-12-17

**Authors:** Yizhou Yao, Haishun Ni, Xuchao Wang, Qixuan Xu, Jiawen Zhang, Linhua Jiang, Bin Wang, Shiduo Song, Xinguo Zhu

**Affiliations:** Department of General Surgery, The First Affiliated Hospital of Soochow University, Suzhou, China

**Keywords:** colorectal cancer, non-invasive screening, intestinal flora, combined detection, fecal immunochemical test (FIT)

## Abstract

**Background:**

The intestinal flora is correlated with the occurrence of colorectal cancer. We evaluate a new predictive model for the non-invasive diagnosis of colorectal cancer based on intestinal flora to verify the clinical application prospects of the intestinal flora as a new biomarker in non-invasive screening of colorectal cancer.

**Methods:**

Subjects from two independent Asian cohorts (cohort I, consisting of 206 colorectal cancer and 112 healthy subjects; cohort II, consisting of 67 colorectal cancer and 54 healthy subjects) were included. A probe-based duplex quantitative PCR (qPCR) determination was established for the quantitative determination of candidate bacterial markers.

**Results:**

We screened through the gutMEGA database to identify potential non-invasive biomarkers for colorectal cancer, including *Prevotella copri* (*Pc*), *Gemella morbillorum* (*Gm*), *Parvimonas micra* (*Pm*), *Cetobacterium somerae* (*Cs*), and *Pasteurella stomatis* (*Ps*). A predictive model with good sensitivity and specificity was established as a new diagnostic tool for colorectal cancer. Under the best cutoff value that maximizes the sum of sensitivity and specificity, *Gm* and *Pm* had better specificity and sensitivity than other target bacteria. The combined detection model of five kinds of bacteria showed better diagnostic ability than *Gm* or *Pm* alone (AUC = 0.861, *P* < 0.001). These findings were further confirmed in the independent cohort II. Particularly, the combination of bacterial markers and fecal immunochemical test (FIT) improved the diagnostic ability of the five bacteria (sensitivity 67.96%, specificity 89.29%) for patients with colorectal cancer.

**Conclusion:**

Fecal-based colorectal cancer-related bacteria can be used as new non-invasive diagnostic biomarkers of colorectal cancer. Simultaneously, the molecular biomarkers in fecal samples are similar to FIT, have the applicability in combination with other detection methods, which is expected to improve the sensitivity of diagnosis for colorectal cancer, and have a promising prospect of clinical application.

## Introduction

In recent years, colonoscopy has been widely used in the diagnosis and screening of colorectal cancer. However, non-invasive screening becomes increasingly important due to the psychological and economic factors of the patients. Although blood/plasma biomarker detection is partially used in some ways, the detection effect is limited, indicating significant differences between studies (Pedlar et al., 2019; Loomba and Adams, 2020). Similarly, only the detection effect of fecal immunochemical test (FIT) is unstable, and its sensitivity and accuracy still need to be further improved (Lee et al., 2014).

With the gradual development of intestinal flora research and the gradual popularization of fecal microbiota transplantation (FMT) and other technologies, the detection technology of intestinal flora has been relatively mature (Paramsothy et al., 2017; Weingarden and Vaughn, 2017; Chen et al., 2019). In the current medical environment, the identification and evaluation of molecular biomarkers in fecal samples is likely more promising than a non-invasive diagnosis of colorectal cancer compared with blood/plasma biomarker tests or FIT testing alone. Abnormal intestinal flora is considered as a potentially important cause of the occurrence and development of colorectal cancer (Kassinen et al., 2007; Gao et al., 2017; Lucas et al., 2017; Wong and Yu, 2019). With the widespread application of metagenomic sequencing and pyrophosphate sequencing in intestinal flora studies, more bacteria have been found positively associated with the occurrence of colorectal cancer (Ahn et al., 2013). In addition, the application of these bacterial candidates in diagnostic biomarkers requires further study in using simple, cost-effective, and targeted methods such as quantitative PCR (qPCR).

In this study, we identified bacterial candidates that may be non-invasive biomarkers of colorectal cancer through gutMEGA database screening, including *Prevotella copri* (*Pc*), *Gemella morbillorum* (*Gm*), *Parvimonas micra* (*Pm*), *Cetobacterium somerae* (*Cs*), and *Pasteurella stomatis* (*Ps*) (Yu et al., 2017). Based on these five bacteria, we have established a predictive model with good sensitivity and specificity for subjects as a new diagnostic tool for colorectal cancer.

## Materials and Methods

The data, analytical methods, and study materials for the purposes of reproducing the results or replicating the procedures can be made available on request to the corresponding authors.

### Collection of Human Stool Samples

Feces samples (*n* = 439) were collected from two separate cohorts: cohort I from 2018 to 2020, The First Affiliated Hospital of Soochow University, 318 subjects, consisting of 206 colorectal cancer patients (average age of 62.2 ± 8.6 years old; with 125 males, 81 females) and 112 normal controls (56.2 ± 12.8 years old; 63 males and 49 females); and cohort II from 2018 to 2020, The First Affiliated Hospital of Soochow University, 121 subjects, consisting of 67 patients with colorectal cancer (mean age of 67.2 ± 9.3 years old; 43 males, 24 females) and 54 normal control groups (56.7 ± 13.5 years old; 30 males, 24 females) (**Supplementary Table S1**). Fecal samples were collected from individuals exhibiting symptoms such as altered bowel habits, rectal bleeding, abdominal pain, or anemia and asymptomatic individuals who were screened for colonoscopy (Yu et al., 2017). When the intestinal flora is restored to the baseline level, the samples should be collected before or 1 month after the colonoscopy. The exclusion criteria were as follows: i) using antibiotics over the past 3 months, ii) taking a vegetarian diet, iii) conducting invasive medical interventions over the past 3 months, and iv) having a history of any cancer or inflammatory bowel disease (Liang et al., 2017). Participants were required to collect fecal samples in standardized containers at home and to store the samples immediately in their home refrigerator at -20°C. The frozen samples were then transported in an insulated polystyrene foam container to the hospital and immediately stored at -80°C until further analysis. The patient was diagnosed by colonoscopy and by a histopathologic examination of any biopsy. Informed consent was obtained from all subjects. The study was approved by the Institutional Review Board (IRB) of the First Affiliated Hospital of Soochow University (2021112).

### DNA Extraction

Fecal samples were thawed on ice and DNA extraction was performed using the QIAamp DNA Stool Mini Kit according to the instructions of the manufacturer (Qiagen). Extracts were then treated with DNase-free RNase to eliminate RNA contamination. DNA quality and quantity were determined using a NanoDrop2000 spectrophotometer (Thermo Fisher Scientific). Primers and probes used in this study are shown in **Supplementary Table S2**.

### Quantitative PCR Reaction

qPCR amplification is performed in a 20-μl reaction system of TaqMan Universal Master Mix II (Applied Biological Systems) containing 0.3 μmol/L of 96-hole reaction sheets and 0.2 μmol/L of reaction sheet seals. The thermal cycler parameters of an ABI PRISM 7900HT sequence detection system were 95°C 10 min × 45 cycles. Each experiment included positive/reference and negative controls (water as a template). Three measurements were repeated for each sample. The qPCR data were analyzed using sequence detection software (Applied Biological Systems) (Liang et al., 2017). Data analysis was performed according to the ΔCq method, using Cq_target_ − Cq_control_ and relative abundance = Power (2, −ΔCq). The nucleotide sequences of primers and probes are shown in **Supplementary Table S2**.

### Feces Immunochemistry Test

Each participant received a Medline iFOB (Medline Industries Inc., Northfield, IL, USA, Lot nom. 768L11) collection tube. Medline iFOB is a qualitative FIT product exempted by the Clinical Laboratory Improvement Act (CLIA). The lower limit of hemoglobin detection set by the manufacturer was 50 g/g. The feces were exposed to ambient temperature for no more than 48 h between emptying and treatment. Each sample was processed in accordance with the instructions of the manufacturer, including verification of activated internal controls. The results of each test were interpreted by two members of the research team (Malagon et al., 2020; Knapp et al., 2021).

### Statistical Analysis

This study used IBM SPSS 25.0 software (SPSS Inc., Chicago, IL, USA) for data statistical analysis and logical regression analysis. Classification data were presented by frequency (percentage), chi-square analysis, or Fisher exact probability method. Continuous data were presented as mean ± standard deviation, and group differences were analyzed using *t*-test or non-parametric Mann–Whitney *U* test. All tests were bilateral, and *P <*0.05 indicated significant statistical differences. In this study, the software R 3.4.3 (http://www.R-project.org, The R Foundation) was used to construct the logistic regression forecasting model, and charts were analyzed using the R software, where the receiver operating feature (ROC) curve was drawn using the Hiplot visual drawing website (https://hiplot.com.cn/). According to the actual flora abundance of the case and predicted situation of the model, the ROC curve was drawn. The area under the curve (AUC) and the differentiation size of the model were also evaluated. The larger the AUC value of the model, the better the prediction effect. Generally, AUC ≥0.8 indicates good differentiation and clinical significance, and AUC <0.6 indicates poor clinical application value. The best truncation value (cutoff value) was determined by the ROC curve and calculated for sensitivity, specificity, diagnostic accuracy, diagnostic ratio, positive prediction rate, positive result likelihood ratio, negative result likelihood ratio at the best cutoff value, and 95% confidence interval. The net gain of the prediction model is evaluated by the predictive model’s decision curve (decision curve analysis, DCA). DCA, as a simple way to evaluate clinical predictive models, diagnostic trials, and molecular markers, is able to better estimate the model for clinical value than AUC by integrating patient or decision-maker preferences into the analysis (Vickers and Elkin, 2006). After determining the best cutoff value of the predictive model, its nomogram was drawn, which can transform the complex regression equation into a visualized graph and make the results of the prediction model more readable, facilitating the evaluation of the patient.

## Results

### Screening of Fecal Bacterial Markers

**Figure 1:**
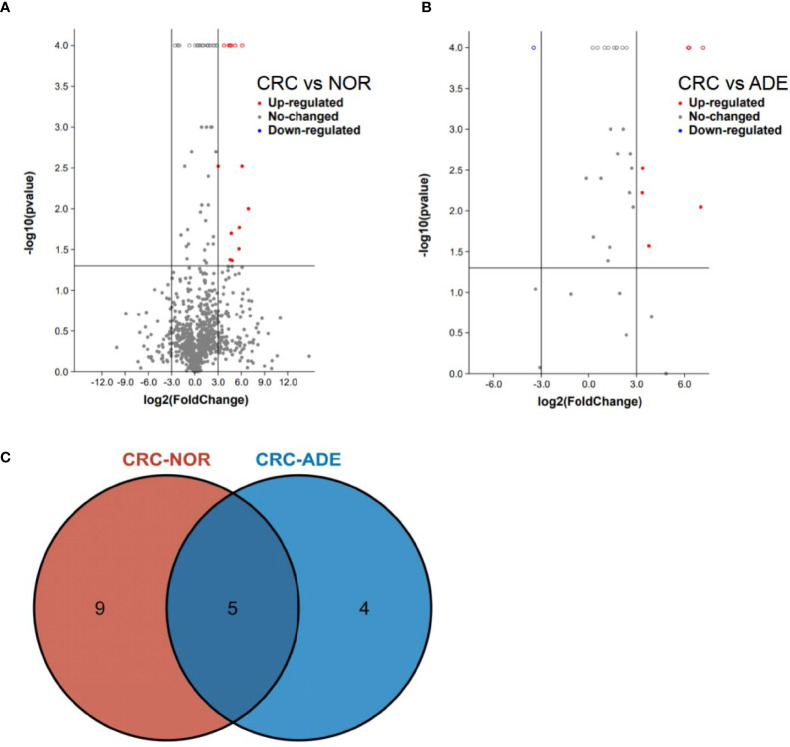
gutMEGA database analysis. **(A)** The volcano chart shows that the species level of colorectal cancer is compared with the normal human intestinal flora, and the species level meets the conditions of Log2(colorectal cancer/NOR) > 3, *P* < 0.05. **(B)** The volcano chart shows that the species level of colorectal cancer patients meets the conditions of Log2(colorectal cancer/ADE) > 3, *P* < 0.05 compared with those with adenoma. **(C)** The Venn diagram shows the analysis results of the gutMEGA database. ADE, adenoma; NOR, normal person.

The progression of enteritis-related bowel cancer and intestinal flora was analyzed through the gutMEGA database (http://gutmega.omicsbio.info/). Log_2_(colorectal cancer/NOR) > 3 and Log_2_(colorectal cancer/ADE) > 3 and *P <*0.05 were set. The associated abundance differences (**Figures 1A, B** **)** were obtained through screening studies. The Venn map showed that there were 13 species of intestinal flora in colorectal cancer and normal human that have significant differences in abundance and 9 species in colorectal cancer and adenoma, where the *Pc*, *Gm*, *Pm*, *Cs*, and *Ps* bacteria achieved statistical differences simultaneously in both groups of controls (Log_2_Ratio > 3, *P* < 0.05, **Figure 1C**).

### *Gm* and *Pm* Can Be Potential Non-Invasive Fecal Biomarkers for Diagnosing Patients With Colorectal Cancer

**Figure 2:**
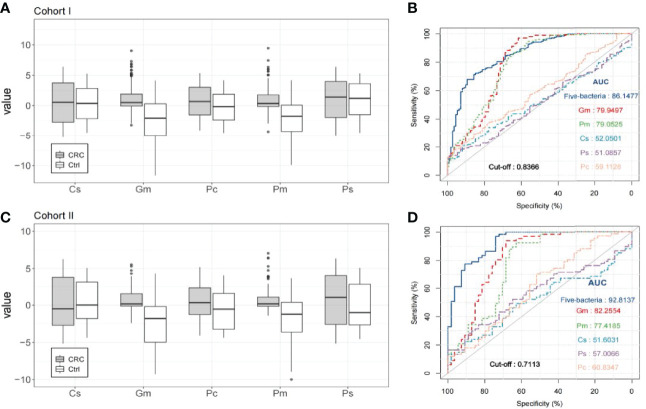
The quantitative detection of fecal bacterial markers in the diagnosis of patients with colorectal cancer (CRC). **(A)** Relative abundance of five bacteria (*Cs*, *Gm*, *Pc*, *Pm*, and *Ps*) in the stool of 206 colorectal cancer patients and 112 healthy subjects in cohort I. **(B)** The receiver operating characteristic (ROC) curve of the markers *Cs*, *Gm*, *Pc*, *Pm*, and *Ps* to identify colorectal cancer patients and healthy controls in cohort I. **(C)** Relative abundance of the five bacteria (*Cs*, *Gm*, *Pc*, *Pm*, and *Ps*) in the stool of 67 colorectal cancer patients and 54 healthy subjects in cohort II. **(D)** The ROC curve of the markers *Cs*, *Gm*, *Pc*, *Pm*, and *Ps* to identify colorectal cancer patients and healthy controls in cohort II.

In all five bacteria, *Gm* and *Pm* performed better in distinguishing colorectal cancer from healthy control than the other three target bacteria, and the relative abundance of *Gm* and *Pm* in colorectal cancer patients was significantly higher than that in healthy control groups (*P* < 0.001; **Figure 2A**). The AUC of *Gm* in cohort I is 0.799 (95% confidence interval of 0.751–0.842; *P* < 0.001; **Figure 2B**) and that of *Pm* in cohort I is 0.791 (95% confidence interval of 0.749–0.838; *P* < 0.001; **Figure 2B**). At the best cutoff value which maximizes the sensitivity and specificity in ROC analysis, *Gm* had a sensitivity of 97.09% to 206 colorectal cancer patients and 112 healthy people, specificity of 61.61%, negative predictive value (NPV) of 92.00%, and positive predictive value (PPV) of 82.30%. The sensitivity of *Pm* to this cohort was 93.20%, specificity was 58.04%, NPV was 82.28%, and PPV was 80.33%. This was further validated in the independent cohort II of 67 colorectal cancer patients and 54 healthy controls. The relative abundance of *Gm* and *Pm* in colorectal cancer patients remained significantly higher than in the healthy control groups (*P* < 0.001; **Figure 2C**). As a single factor which distinguishes colorectal cancer patients and the control groups, the AUC of the fecal biomarker candidate *Gm* was 0.823 (0.762–0.893, *P* < 0.001, **Figure 2D**), and that of Pm was 0.774 (0.712–0.838, *P* < 0.001, **Figure 2D**).

### The Combination of *Gm*, *Pm*, *Pc*, *Cs*, and *Ps* Improves the Ability of *Gm* and *Pm* to Diagnose Patients With Colorectal Cancer

**Figure 3:**
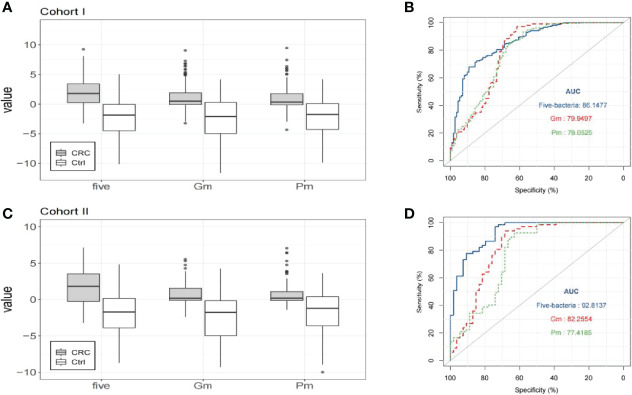
The combination of five markers can improve the diagnostic ability of colorectal cancer (CRC). **(A)** Compared with the healthy control group of cohort I, the relative stool abundance of *Gm*, *Pm*, and five bacteria (*Gm*, *Pm*, *Pc*, *Ps*, *Cs*) in colorectal cancer patients. **(B)** The ROC curve of the five selected candidate bacterial markers in cohort I, including the simple linear combination of *Prevotella copri* (*Pc*), *Gemella morbillorum* (*Gm*), *Parvimonas micra* (*Pm*), *Cetobacterium somerae* (*Cs*), and *Pasteurella stomatis* (*Ps*). **(C)** Compared with the healthy control group of cohort II, the relative stool abundance of *Gm*, *Pm*, and five bacteria (*Gm*, *Pm*, *Pc*, *Ps*, *Cs*) in colorectal cancer patients. **(D)** The ROC curve of the five selected candidate bacterial markers in cohort II, including the simple linear combination of *Prevotella copri* (*Pc*), *Gemella morbillorum* (*Gm*), *Parvi monasmicra* (*Pm*), *Cetobacterium somerae* (*Cs*), and *Pasteurella stomatis* (*Ps*).

In line with the data after sequencing, the combined use of the bacterial markers tested showed better diagnostic performance than *Gm* or *Pm* alone, with AUC increasing to 0.8615 (**Figure 3B**). It was found that the simple linear combination of *Gm*, *Pm*, *Pc*, *Ps*, and *Cs* had higher AUC (0.862) than other combinations (two to four markers) or *Gm* (0.799) or *Pm* (0.791) alone. The relative abundance of the five bacteria in patients with colorectal cancer was significantly higher than that in healthy control groups (*P* < 0.001, **Figure 3A**). Under the best cutoff value, the five bacteria (*Gm*, *Pm*, *Pc*, *Ps*, *Cs*) can distinguish patients with colorectal cancer from healthy controls, with a sensitivity of 67.96%, specificity of 89.29%, NPV of 60.24%, and PPV of 92.11%, which show better diagnostic performance than *Gm* or *Pm* alone (**Table 1**). The pairing comparison of AUC showed that the colorectal cancer diagnostic ability of the five-bacteria group was better than that of the group *Gm* or *Pm* alone (*P* < 0.05).

**Table 1:** Performance of *Gm* and *Pm* alone and in combination with other bacteria for the diagnosis of colorectal cancer in cohort I.

Variable	*Gm*	*Pm*	Combination
AUC	0.7995	0.7905	0.8614
Cutoff	−1.5948	−1.0548	0.8366
Sensitivity	97.09%	93.20%	67.96%
Specificity	61.61%	58.04%	89.29%
PPV	82.30%	80.33%	92.11%
NPV	92.00%	82.28%	60.24%

The best cutoff value that maximizes sensitivity and specificity is used.

AUC, area under the receiver operating characteristic curve; NPV, negative predictive value; PPV, positive predictive value.

The diagnostic performance of the five bacteria was further demonstrated in the independent cohort II. The combination of the five bacteria also showed an increase in AUC (0.928, **Figure 3D**). In patients with colorectal cancer, the relative abundance of the five bacteria was significantly higher than that in the healthy control group (*P* < 0.001, **Figure 3C**). It showed better diagnostic performance in the combination of the five bacteria than *Gm* or *Pm* alone. Therefore, the combination of *Gm*, *Pm*, *Pc*, *Cs*, and *Ps* improves the diagnostic ability of *Gm* and *Pm* to identify colorectal cancer and healthy control groups.

### The Combined Application of Bacterial Markers and FIT Improves the Diagnostic Ability of Bacteria Alone for Patients With Colorectal Cancer

**Figure 4:**
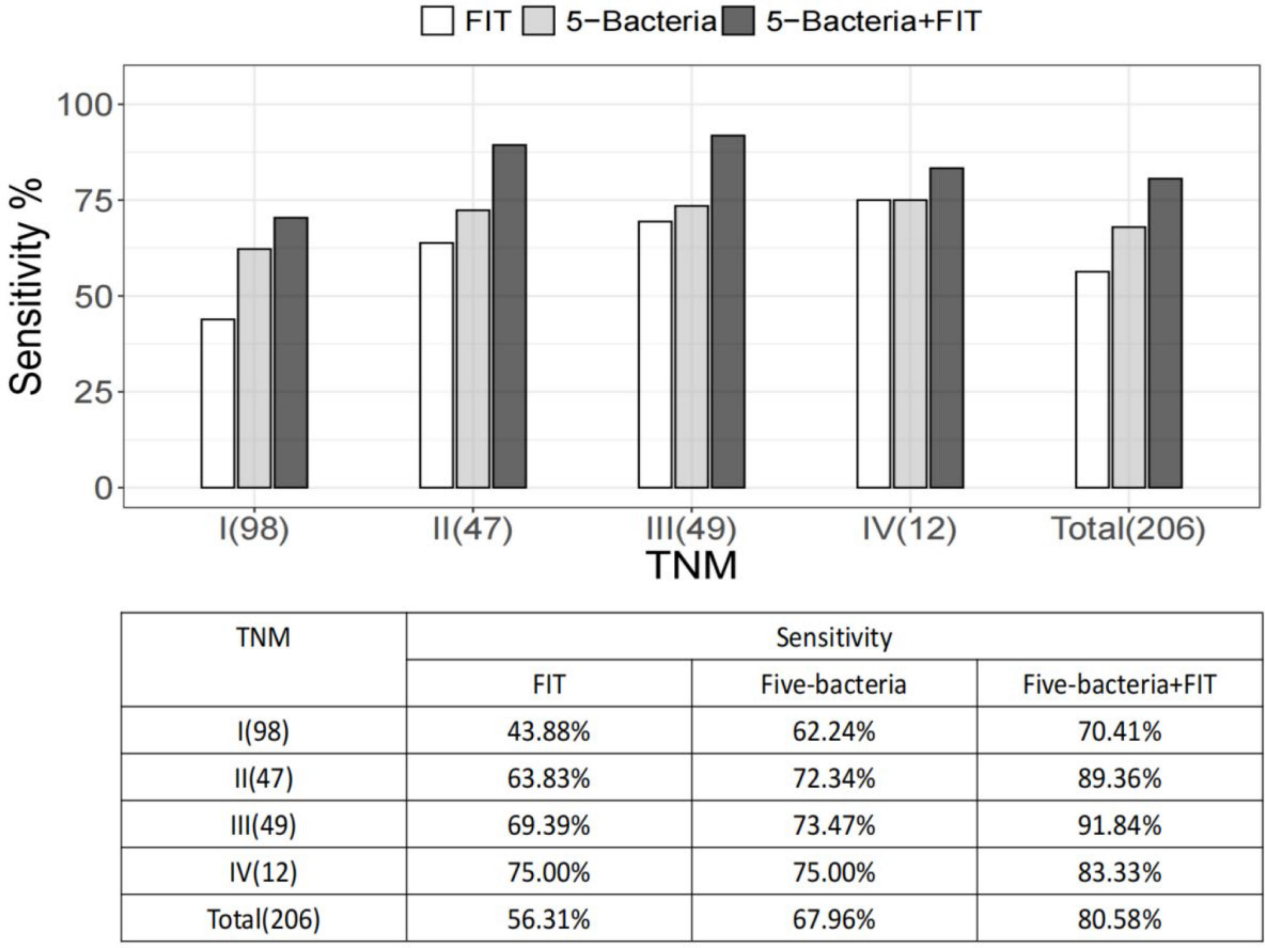
Commercial fecal immunochemical test (FIT) and sensitivity to bacterial markers based on a subset of tumor-nodal-metastasis (TNM) stages. Shown also is the sensitivity of FIT and four bacteria, combined with the detection of colorectal cancer based on the tumor stage of colorectal cancer. The numbers in parentheses are the number of participants in each category.

FIT stool specimens were tested from 206 patients with colorectal cancer and 112 normal controls in cohort I. We found that 56.31% of the stool specimens from colorectal cancer patients were FIT positive. In this subcohort of colorectal cancer patients, the detection rate of FIT was lower than the combination of five bacteria (67.96%, *P* < 0.05). Pairwise comparison of the ROC curve showed that the combinations of five bacteria were significantly superior to *Gm* or *Pm* alone for the diagnosis of colorectal cancer (both *P* < 0.05). FIT was slightly related with TNM staging (*P* = 0.076), while the relative abundances of the five bacteria or *Gm* and *Pm* were independent of TNM staging. Assessment of colorectal cancer detection in the TNM staging subgroup showed that the quantification of bacterial markers was much more sensitive than FIT in stage I. Similarly, in stage II and III, we observed more prominent detection rates by bacteria than FIT (**Figure 4**). It indicated that the sensitivity of the quantitative detection bacterial markers is significantly higher than FIT, especially for non-metastatic colorectal cancer.

After the combination of bacterial markers and FIT, the sensitivity of FIT increased from 56.31% to 80.58% and the five-bacteria group from 67.96% to 80.58%, PPV and NPV were both improved, and the specificity was almost unchanged (**Table 2**). According to the I–IV stages of TNM staging, the sensitivity of the binding of bacterial markers to FIT was significantly higher than FIT alone. It suggested that the joint detection of bacterial markers and FIT had elevated sensitivity and specificity for the non-invasive diagnosis of colorectal cancer.

**Table 2:** Performance of FIT alone, combined with five bacteria to diagnose colorectal cancer in cohort I.

Variable	FIT	Five bacteria	Five bacteria + FIT
Sensitivity	56.31%	67.96%	80.58%
Specificity	94.64%	89.29%	87.50%
PPV	95.08%	92.11%	92.22%
NPV	54.08%	60.24%	71.01%

NPV, negative predictive value; PPV, positive predictive value.

### Significant Clinical Benefits of the Colorectal Cancer Risk Prediction Model Based on the Five-Bacteria Combination

**Figure 5:**
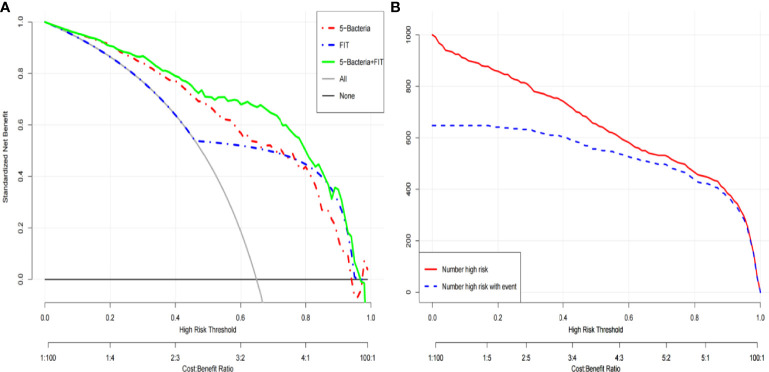
The clinical decision curve **(A)** and clinical impact curve **(B)** of the colorectal cancer risk prediction model combined with FIT.

**Figure 6:**
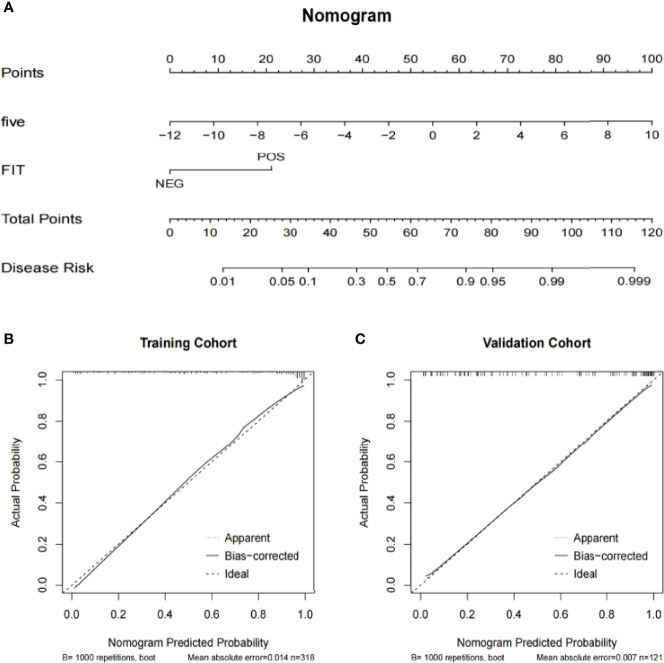
The nomogram of the risk prediction models for colorectal cancer. **(A)** Nomogram of the colorectal cancer risk prediction model combined with FIT. **(B)** The training cohort prediction calibration curve of the colorectal cancer risk prediction model combined with FIT. **(C)** The validation cohort prediction calibration curve of the colorectal cancer risk prediction model combined with FIT.

To further demonstrate the clinical application prospects of the five target bacteria, cohort I was used as the training group to construct the colorectal cancer risk model, and cohort II was analyzed as the testing group to test the feasibility of the cohort I-dependent testing model (Dong et al., 2019; Sun et al., 2019). The clinical decision curve and clinical impact curve of the colorectal cancer risk prediction model combined with FIT showed a significant clinical benefit of the prediction model (**Figures 5A, B** **)**. To facilitate the clinical application of the colorectal cancer risk prediction model for the combination of five bacteria, we further constructed a nomogram based on this model (**Figure 6A**). The relative abundance of the five bacteria combinations and FIT was assigned, and the total score was calculated to determine the likelihood of complications. When the total points are up to 50, the predicted model predicts a probability of 0.4 (**Figure 6A**). Taking the prediction of the possibility of colorectal cancer as the transverse coordinate and the actual positive rate of the case as the longitudinal coordinate, the prediction calibration curve (calibration curve) of the model showed the overall prediction with the actual situation (**Figures 6B, C** **)**. The prediction accuracy of the model was not significantly reduced with the change of the population, which proved the reliability of the colorectal cancer risk model based on the five target bacteria.

## Discussion

In recent years, colonoscopy has been widely used for colorectal cancer diagnosis and screening. However, non-invasive screening becomes increasingly important due to the psychological and economic factors of the patients. With the gradual development in the study of intestinal microbiota and the gradual promotion of FMT and other technologies, the detection technology of intestinal microbiota has been relatively mature.

The occurrence and development of many clinical diseases are closely related to the change of microbiota abundance (Garrett, 2015; Valdes et al., 2018). The application of metagenomic sequencing in the field of microorganisms has further revealed the changes in the diversity and abundance of bacterial communities during disease progression. Among them, extensive research on colorectal cancer has led to the identification of the bacteria associated with colorectal cancer (Jiang et al., 2017; Mangiola et al., 2018; Nishida et al., 2018; Wang and Zhao, 2018). However, it is an important issue to choose from the identified bacteria with different abundance, establish a stable and efficient clinical detection model, and evaluate their clinical application potential. According to the analysis of existing studies, it is currently more feasible to detect the target intestinal microbiota in fecal samples by qPCR due to the reliable correlation between metagenomic sequencing and qPCR detection of the target bacteria (Liang et al., 2017; Pittman, 2018). However, the combination of target bacteria included in the detection model varies greatly between different studies. Additionally, the modeling and testing evaluation of qPCR detection results are still not reliable.

Target bacteria (*Pc*, *Gm*, *Pm*, *Cs*, *Ps*) with significant changes in abundance during the tumorigenesis of colorectal cancer were searched through the gutMEGA platform (Zhang et al., 2021). Strict inclusion requirements were developed so that detection of the target bacteria based on screening would not be affected by common lifestyle factors known to influence the composition of the gut microbiota. Antibiotics, proton pump inhibitors, metformin, and painkillers, for example, may alter the composition of the gut flora (Rogers and Aronoff, 2016; Zhernakova et al., 2016). Participants included in this study were required to explicitly exclude the effects of these factors to limit the confounding effects of these variables. However, the potential bias caused by these factors remains a major challenge for non-invasive diagnostic platforms based on gut microbiota for some time.

According to the inclusion criteria, cohort I included and detected the target bacteria in the fecal samples of 318 subjects, consisting of 206 patients with colorectal cancer and 112 healthy people. The specificity and sensitivity of *Gm* and *Pm* were significantly better than other target bacteria. The combined detection of the five bacteria showed the largest AUC area, indicating that the combined detection effect of five bacteria was better than that of a single bacteria, which proved that the combined detection of these five bacteria had clinical diagnostic significance and potential application prospect. Stool samples from 67 colorectal cancer patients and 54 healthy people were collected in cohort II, and the detection results were consistent with the trend of cohort I, which proved the stability and feasibility of this combination model.

The detection model composed of five target bacteria was more sensitive to the diagnosis of colorectal cancer than FIT test alone. With the gradual increase of TNM stage in colorectal cancer patients, the diagnostic sensitivity of the target bacteria was elevated. However, the diagnostic sensitivity of TNM stage IV patients was lower than that of TNM stage III patients, which may result from statistical bias caused by the low sample size of stage IV patients (Rao, 2012). Interestingly, we found that the results of the FIT test in some patients were not consistent with the results of bacterial assessment, which provided the possibility for the complementarity of the two methods. Therefore, we evaluated the diagnostic efficacy of the target bacteria test combined with the FIT test and found an obvious improvement in sensitivity. It indicates that molecular biomarkers in fecal samples as a non-invasive detection method, similar to FIT, have the applicability in combination with other detection methods, which is expected to improve the sensitivity of diagnosis and have a promising prospect of clinical application.

In order to further demonstrate the clinical application prospect of the five target bacteria included, cohort I patients were taken as the training group to construct a colorectal cancer risk model (Dong et al., 2019; Sun et al., 2019). Cohort II patients were taken as the testing group to test the feasibility of the cohort I-dependent detection model. The results showed a positive test, and the prediction accuracy was not significantly reduced by changing the test population, demonstrating the reliability of the colorectal cancer risk model based on the five target bacteria.

In previous research, *Fusobacterium nucleatum* (*Fn*) is generally regarded as an oral pathogen, and there is increasing evidence that *Fusobacterium* infection is common in colorectal carcinoma. It may be a promising biomarker that is predictive of the clinical outcome in patients with CRC (Castellarin et al., 2012; Guo et al., 2018; Yamaoka et al., 2018). In order to discover more intestinal flora related to colorectal cancer and explore its application prospects, we screened through the gutMEGA database and identified candidate bacteria that may be non-invasive biomarkers for colorec\tal cancer in this study. To sum up, in this study, the combination of bacterial markers (*Pc*, *Gm*, *Pm*, *Cs*, *Ps*) and FIT improved the diagnostic ability for CRC patients, which has broad application prospects and is worthy of further study.

Validation of potential microbial markers in a large group of people is an important means of non-invasive screening of colorectal cancer in the general population, with high requirements for accuracy, stability, and practicability (Zeller et al., 2014; Pittman, 2018). Although microbial markers show great potential in the current diagnosis of non-invasive colorectal cancer, problems and challenges still exist in combining with or replacing traditional non-invasive tests (Silbergeld, 2017; Shah et al., 2018). Hence, improving the feasibility of a microbial marker detection model gradually and making it an effective supplement to non-invasive screening of colorectal cancer may be an important way to develop a microbial marker-based diagnosis of colorectal cancer.

## Data Availability Statement

The original contributions presented in the study are included in the article/**Supplementary Material**. Further inquiries can be directed to the corresponding authors.

## Ethics Statement

The studies involving human participants were reviewed and approved by the Ethics Committee of the First Affiliated Hospital of Soochow University. The patients/participants provided their written informed consent to participate in this study.

## Author Contributions

XZ and SS conceived and supervised the project. YY, HN, and XW contributed to the experimental design, prepared the figures, and wrote the manuscript. All authors performed the experiments and interpreted the results. All authors discussed the results and approved the manuscript.

## Funding

This work was supported by the National Nature Science Foundation of China (81974375) and Project of National Science Foundation of Jiangsu Province (BK20161225).

## Conflict of Interest

The authors declare that the research was conducted in the absence of any commercial or financial relationships that could be construed as a potential conflict of interest.

## Publisher’s Note

All claims expressed in this article are solely those of the authors and do not necessarily represent those of their affiliated organizations, or those of the publisher, the editors and the reviewers. Any product that may be evaluated in this article, or claim that may be made by its manufacturer, is not guaranteed or endorsed by the publisher.

## Supplementary Material

Supplementary Table S1 Clinical characteristics of healthy subjects and colorectal cancer patients. colorectal cancer, colorectal cancer; BMI, body mass index; TNM, tumor-node-metastasis. Gender by Chi-square; Age and BMI by T-test.

Supplementary Table S2 Primers and probes used in this study.
