# Supplementary material for: A New Biomarker of Fecal Bacteria for Non-Invasive Diagnosis of Colorectal Cancer
Source: Front Cell Infect Microbiol. 2021 Dec 17;11:744049. doi: 10.3389/fcimb.2021.744049 (PMC8719628; doi:10.3389/fcimb.2021.744049)
Supplement: Supplementary Table S1 — Clinical characteristics of healthy subjects and colorectal cancer patients. colorectal cancer, colorectal cancer; BMI, body mass index; TNM, tumor-node-metastasis. Gender by Chi-square; Age and BMI by T-test. [file Table_1.docx]

**Supplementary Table S1.** Clinical characteristics of healthy subjects and colorectal cancer patients

|  | Cohort I | | | Cohort II | | |
| --- | --- | --- | --- | --- | --- | --- |
| Variables | Colorectal cancer  (n=206) | Control  (n=112) | P value | Colorectal cancer  (n=67) | Control  (n=54) | P value |
| Age | 62.2 ± 8.6 | 56.2 ± 12.8 | <0.0001 | 67.2±9.3 | 56.7±13.5 | <0.0001 |
| Gender |  |  |  |  |  |  |
| Male | 125 (60.7%) | 63 (56.3%) | 0.443 | 43 (64.2%) | 30 (55.6%) | 0.335 |
| Female | 81 (39.3%) | 49 (43.7%) |  | 24 (35.8%) | 24 (44.4%) |  |
| BMI | 21.4±2.2 | 22.8±2.4 | <0.001 | 21.2±2.3 | 22.5±2.1 | <0.001 |
| TNM stage |  |  |  |  |  |  |
| I | 98 (47.6%) |  |  | 13 (19.4%) |  |  |
| II | 47 (22.8%) |  |  | 22 (32.8%) |  |  |
| III | 49 (23.8%) |  |  | 23 (34.3%) |  |  |
| IV | 12 (5.8%) |  |  | 9 (13.4%) |  |  |

Notes: colorectal cancer, colorectal cancer; BMI, body mass index; TNM, tumor-node-metastasis. Gender by Chi-square; Age and BMI by T-test.

**Supplementary Table S2.** Primers and probes used in this study

| Targets |  | Nucleotide sequence (5'->3') | Size (bp) |
| --- | --- | --- | --- |
| Prevotella copri | Forward | CGCGAACTGGTTTCCTTGA | 56 |
|  | Reverse | ACCGCTACACCACGAATTCC |  |
|  | Probe | FAM-ACGCACAAAGTGGG-MGB |  |
| Gemella morbillorum | Forward | AGATGGCTTTGCGGTGCATT | 140 |
|  | Reverse | ATTCCCTACTGCTGCCTCCC |  |
|  | Probe | FAM-AAAGGCCCACCAAGGCGACGATGCA-MGB |  |
| Parvimonas micra | Forward | TCGAACGTGATTTTTGTGGAAA | 102 |
|  | Reverse | GGTAGGTTGCTCACGTGTTACTCA |  |
|  | Probe | FAM-CCCGTTCGCCACTT-MGB |  |
| Cetobacterium somerae | Forward | TGCAAGTCGAACGGTAGCAG | 113 |
|  | Reverse | GCTGCAGTTTCCCGCAGTTA |  |
|  | Probe | FAM-TTCTTGCTGACGAGTGGCGACGGGT-MGB |  |
| Pasteurella stomatis | Forward | AGAGAGAGCCTTGCGTTCCA | 115 |
|  | Reverse | TAAGGGCCGTGTCTCAGTCC |  |
|  | Probe | FAM-AACGGCCCACCAAGGCGACGATGGATA-MGB |  |
